# Effects of monofilament nylon versus braided multifilament nylon gangions on catch rates of Greenland shark (*Somniosus microcephalus*) in bottom set longlines

**DOI:** 10.7717/peerj.10407

**Published:** 2020-12-03

**Authors:** Scott M. Grant, Jenna G. Munden, Kevin J. Hedges

**Affiliations:** 1 Centre for Sustainable Aquatic Resources, Memorial University of Newfoundland, St. John’s, NL, Canada; 2 Herring Science Council, Halifax, NS, Canada; 3 Arctic Aquatic Research Division, Central & Arctic Region, Fisheries and Oceans Canada, Winnipeg, MB, Canada

**Keywords:** Greenland shark, Greenland halibut, Bycatch mitigation, Longline, Conservation, Monofilament gangion, Entanglement, Cumberland sound, Circle hooks

## Abstract

The Greenland shark (*Somniosus microcephalus*) is the main bycatch species in established and exploratory inshore longline fisheries for Greenland halibut (*Reinhardtius hippoglossoides*) on the east coast of Baffin Island, Canada. Bycatch and entanglement in longline gear has at times been substantial and post-release survival is questionable when Greenland sharks are released with trailing fishing gear. This study investigated the effect of the type of fishing line used in the gangion and gangion breaking strength on catch rates of Greenland shark and Greenland halibut in bottom set longlines. Circle (size 14/0, 0° offset) hooks were used throughout the study. Behavior of captured sharks, mode of capture (i.e., jaw hook and/or entanglement), level of entanglement in longline gear, time required to disentangle sharks and biological information (sex, body length and health status) were recorded. Catch rates of Greenland shark were independent of monofilament nylon gangion breaking strength and monofilament gangions captured significantly fewer Greenland sharks than the traditional braided multifilament nylon gangion. Catch rates and body size of Greenland halibut did not differ significantly between gangion treatments. Although most (84%) of the Greenland sharks were hooked by the jaw, a high percentage (76%) were entangled in the mainline. The mean length of mainline entangled around the body and/or caudal peduncle and caudal fin was 28.7 m. Greenland sharks exhibited cannibalistic behavior with 15% of captured sharks cannibalized. All remaining sharks were alive and survived the disentanglement process which can be attributed to their lethargic behavior and lack of resistance when hauled to the surface. Thus, as a conservation measure fishers should be encouraged to remove trailing fishing gear prior to release. Our results are used to demonstrate benefits to the fishing industry with regard to an overall reduction in the period of time to disentangle sharks and damage to fishing gear by switching from braided multifilament to monofilament gangions in Greenland halibut longline fisheries.

## Introduction

In 1986, a small scale winter (January–May) longline fishery for Greenland halibut (*Reinhardtius hippoglossoides*) was developed in Cumberland Sound, a large inlet on the east coast of Baffin Island in the territory of Nunavut, Canada. In this fishery, fishing takes place through holes in the ice and fishers move with land-fast ice as it expands to cover deeper waters (>400 m) where Greenland halibut are abundant. Local interest from the indigenous community of Pangnirtung grew rapidly and in 1994 a new fishery management area with a 500 t quota was established in the northern end of Cumberland Sound, where the winter fishery occurs (Fisheries & Oceans Canada, 2008a). Participation (115 fishers) and landings (430 t) peaked in the early to mid-1990s. However, increasingly shorter sea-ice seasons, less stable ice conditions, and loss of gear during winter storms contributed to a substantial reduction in participation and landings with as few as six fishers and 3 *t* harvested in the early 2000’s (Dennard et al., 2010; Fisheries & Oceans Canada, 2008a). Improved ice conditions and catch rates in recent years have resulted in the full 500 *t* quota captured through the ice in some years. However, given the economic importance of the Greenland halibut resource to Inuit in Pangnirtung (ca. $1.9 million annually) and likelihood of a climate change induced return to ice conditions experienced in the early 2000’s, a supplemental longline fishery during the ice-free season (July–October) was proposed. No bottom gillnetting or trawling is permitted in Cumberland Sound (Fisheries & Oceans Canada, 2008b) and future development of a Greenland halibut longline fishery during the ice-free season would increase the likelihood of catching the full quota in years when ice conditions limit winter fishing activities.

Bycatch in the Cumberland Sound winter fishery and exploratory winter longline fisheries for Greenland halibut on the east coast of Baffin Island is primarily Greenland shark (*Somniosus microcephalus*) and to a lesser extent thorny skate (*Amblyraja radiata*) (Fisheries & Oceans Canada, 2008a; Walsh, 2008). Because of their large body size Greenland sharks account for the greatest bycatch by weight. Greenland sharks were also found to account for the highest percentage of the bycatch during a Greenland halibut longline test fishery that took place in Cumberland Sound during the ice-free season in 2009 (Young, 2010). A total of 570 Greenland sharks were captured incidentally (0.63 sharks per 100 hooks) during the test fishery and bycatch of Greenland shark was estimated to be 4.8× the biomass of Greenland halibut landed (Young, 2010).

Hooked Greenland sharks tend to roll and become entangled within bottom longline gear and entanglement can at times be severe (Pike, 1994; Young, 2010; Grant, Sullivan & Hedges, 2018). Consequently, entangled Greenland sharks have often been killed by fishers (Idrobo, 2008) or fishers release entangled sharks with trailing fishing gear (S. Grant, 2014, 2015 personal observation), which has been shown to cause low post-release survival in some elasmobranchs, for example, the common thresher shark (*Alopias vulpinus*) (Sepulveda et al., 2015). Young (2010) indicated that about 50% of the Greenland sharks captured in the 2009 Cumberland Sound open water test fishery were released alive but post-release survival is unknown.

Sharks are known to exhibit a k-selected life history (Hoening & Gruber, 1990), which includes slow growth, long life span, low reproductive potential and late sexual maturity. Recent aging studies of Greenland sharks captured in Arctic waters suggest this species is the longest lived vertebrate (at least 272 years) and age at sexual maturity in females is at least 156 years (Nielsen et al., 2016), making this species particularly vulnerable to exploitation. The Greenland shark is the only species of shark to occur in Arctic waters year round and it is the largest fish species in the Arctic Ocean (Compagno, 1984). Greenland shark is a generalist benthic and pelagic feeder in Arctic waters, it is a known scavenger, and a top predator, consuming fish and marine mammals (Ridoux et al., 1998; Fisk et al., 2002; Yano, Stevens & Compagno, 2007; Idrobo, 2008; McMeans et al., 2010; Leclerc et al., 2011, 2012; MacNeil et al., 2012). As top predators, sharks can play an important role in the ecosystem by affecting populations of their prey species, they help to remove the weak and the sick, and they serve as indicators of ocean health (Strid et al., 2007; McMeans et al., 2010; Courtney & Foy, 2012). Although studies are beginning to obtain a greater understanding of the diet, life history, and general biology of the Greenland shark, there are still uncertainties with regard to their past and present role in the Arctic ecosystem or how their role will be influenced by climate change.

In addition to potential ecological impacts, the bycatch of the Greenland shark has a number of economic costs to longline fishers. When Greenland sharks become entangled in longline gear they can cause damage or loss of gear (Pike, 1994) and considerable time can be spent fully disentangling sharks (Grant, Sullivan & Hedges, 2018). Further, hooks that become entangled by sharks are unlikely to continue to lure and capture Greenland halibut (Dennard et al., 2010). There are clear economic incentives to reducing the bycatch of Greenland shark in bottom longline fisheries. However, identifying a means of reducing bycatch without negatively affecting catch rates and average body size of captured Greenland halibut will be key to gaining support from the fishing industry.

Sustainable exploitation of fishery resources involves identifying ways to preserve the unique Arctic ecology and there is a need to manage Greenland shark bycatch (Food & Agriculture Organization of the United Nations (FAO), 1999; Davis et al., 2013). Methods to reduce the bycatch of Greenland shark include avoidance through a greater understanding of spatial-temporal geographic distribution and gear modifications to reduce the capture or facilitate escapement of sharks. Much of our understanding of the seasonal distribution, movements, and abundance of the Greenland shark or factors influencing these attributes in Canadian Arctic waters are unclear owing in part to a limited ice-free season and the variable deep water benthic and pelagic residency of Greenland sharks (Skomal & Benz, 2004; Stokesbury et al., 2005; Fisk, Lyderson & Kovacs, 2012; Campana, Fisk & Klimley, 2015). For example, all 10 of the Greenland sharks captured through the ice on longlines and fitted with archival satellite pop-off tags in Cumberland Sound during the spring of 2008 appeared to exhibit highly directional long-range (315–1,615 km) spring-summer movements out of Cumberland Sound and northward into Baffin Bay (Campana, Fisk & Klimley, 2015). In 2015, Hussey et al. (2018) also found what appeared to be highly directional long-range (414–617 km) movement in late summer-early autumn by five Greenland sharks tagged with mark-report and archival satellite pop-off tags in Steiness Fjord, located on the south coast of Ellesmere Island, Canada. Eastward movement by these sharks to northwest Greenland and the northward movement into Baffin Bay by sharks tagged in Cumberland Sound by Campana, Fisk & Klimley (2015) led Hussey et al. (2018) to suggest a seasonal migration to overwintering grounds in the North Water Polynya (NWP), an open water region between Jones Sound and northwest Greenland. The NWP may represent overwintering grounds for many Arctic predators (Heide-Jorgensen et al., 2012) however, Cumberland Sound contains its own large recurrent polynya that may also be expected to attract many Arctic predators (Stirling & Cleator, 1981). Further, this seasonal migration hypothesis does not account for the late summer-early autumn capture of 570 Greenland sharks in Cumberland Sound in 2009 (Young, 2010) or the capture of Greenland sharks in the Cumberland Sound winter longline fishery and exploratory winter longline fisheries along the east coast of Baffin Island (Pike, 1994; Walsh, 2008; Fisheries & Oceans Canada, 2008b). Catches of Greenland sharks in the Cumberland Sound winter longline fishery have at times been substantial enough to cause indigenous fishers to reduce longline soak times from 8–10 h to 1–2 h to avoid capturing Greenland sharks (Pike, 1994). In addition, over winter catch rates of Greenland sharks in Scott Inlet (0.69 sharks per 100 hooks; Walsh, 2008), located on the east coast of Baffin Island, were comparable to that reported by Young (2010) in Cumberland Sound during the ice-free season. Greenland sharks tagged in Cumberland Sound were considered immature, measuring <3 m in length (Campana, Fisk & Klimley, 2015) and Grant, Sullivan & Hedges (2018) found that this size class dominated longline catches in Cumberland Sound during late summer, ruling out a life stage explanation for the observed long-range offshore movements reported by Campana, Fisk & Klimley (2015).

From a conservation perspective, a greater understanding of factors influencing the distribution, movements, and local abundance of Greenland shark can help to reduce vulnerability to fishing operations through altered fishing seasons and fishing areas. However, when altered fishing strategies are not feasible, ineffective, or unambiguous information is lacking to make decisions, gear modifications can be used to reduce incidental capture rates of sharks (Stone & Dixon, 2001; Yokota, Kiyota & Minami, 2006; Gilman et al., 2008; Ward et al., 2008; Vega & Licandeo, 2009; Afonso et al., 2012).

Albeit anecdotal, fishers in the Cumberland Sound Greenland halibut winter longline fishery indicate there was a reduction in catch rates and entanglement of Greenland sharks when they switched from J-hooks to circle hooks (Pike, 1994). More recently, circle hooks were found to outperform EZ-hooks with regard to catch rates of Greenland halibut (Woll et al., 1998), and circle hooks have been adopted in Canada’s inshore and offshore Greenland halibut longline fisheries throughout Baffin Bay and Davis Strait (i.e., NAFO Subarea 0). Circle hooks tend to lead to hooking in the jaw rather than throat or gut hooking (Kerstetter & Graves, 2006; Afonso et al., 2011; Pacheco et al., 2011) and this may in part at least account for the apparent decrease in catch rates and entanglement of Greenland shark in the Cumberland Sound winter fishery. Magnetic alloys and electropositive metals have been found to deter feeding and repel some species of sharks (O’Connell et al., 2010; Jordan, Mandelman & Kajiura, 2011; Robbins, Peddemors & Kennelly, 2011). However, magnetized circle hooks that were also treated with an electropositive metal did not deter Greenland shark from feeding on bottom set longlines in Arctic waters (Grant, Sullivan & Hedges, 2018).

The Greenland shark is one of the largest of all shark species with a confirmed maximum length of 640 cm (Bigelow & Schroeder, 1948). However, most reported lengths from fishing gear range from 288 cm to 504 cm (Bigelow & Schroeder, 1953; Fisk et al., 2002) which according to the weight-length equation provided by MacNeil et al. (2012) corresponds to a weight range of 251–1,446 kg. Entanglement of Greenland sharks in bottom longline gear can occur when a shark becomes hooked and begins to roll or when a hooked shark drags the fishing gear along the seabed causing entanglement of the mainline around the body and/or tail region of the shark followed by rolling. The 251 kg estimated weight for the smallest Greenland sharks typically captured in fishing gear is an order of magnitude higher than the maximum weight reported for Greenland halibut (Scott & Scott, 1988). Given the greater weight of Greenland shark and hence greater force exerted on a fishing line it would appear that the simplest gear modification to reduce bycatch in the Greenland halibut longline fishery may be to reduce the breaking strength of the fishing line used in the gangion (see also review by Werner et al., 2006). This would facilitate breakage of the gangion as a hooked shark drags the mainline over the seabed. In addition, the type of fishing line used in the gangion may also be important. Currently braided multifilament nylon is the material used in gangions throughout Canada’s Greenland halibut longline fisheries. At a given breaking strength monofilament nylon fishing line is thinner than braided multifilament nylon. Being thinner and a single strand, monofilament fishing line is expected to chafe and sever more readily when rubbing against placoid scales on the head and body of Greenland sharks, and to sever more readily when contacting a sharks’ teeth, allowing sharks to escape prior to haul back.

This study investigated the effect of varying the type of fishing line used in the gangion (i.e., monofilament nylon versus braided multifilament nylon) and gangion breaking strength on catch rates and the size distribution of captured Greenland shark and Greenland halibut. In addition, relevant biological information on Greenland shark, mode of capture, level of entanglement, time required to safely disentangle and release sharks, and time required to disentangle the mainline once a shark was released were also recorded.

## Materials and Methods

### Experimental longlines

Bottom longline catches of Greenland shark and Greenland halibut were compared among three experimental monofilament nylon gangion treatments of differing breaking strength and a traditional braided multifilament nylon gangion treatment (Table 1). The multifilament fishing line used was Ashaway^®^ tuna line comprised of nine braided nylon strands. The monofilament fishing line used was Momoi^®^ Hi-catch nylon line. The mainline (i.e., groundline) of the experimental longline was made of braided and tarred polyester measuring 14 mm in diameter and was fitted with rotor swivels at 1.8 m intervals. The experimental longline consisted of 400 gangions with 100 each of the traditional braided multifilament nylon and three experimental monofilament nylon gangion treatments. To ensure equal representation of gangion treatments across the gear, the gangions were arranged along the mainline in alternating groups of 20. The pattern of alternation was initially randomized and then remained consistent throughout the experiments (i.e., C-91BN, E-23M, E-91M and E-46M). Gangion treatments were identified by attaching colour-coded twine at each end of a 20 hook replicate. Mustad^®^ carbon steel (i.e., duratin) circle 14/0 hooks with 0° offset and 15.4 mm gap were used throughout the experimental longline. All hooks were baited with previously frozen squid cut into 11–20 cm length pieces (mean, 15.2 cm). All hooks were baited by hand which ensured 100% of the hooks were baited. The gear was observed to pay-out of longline tubs and into the ocean with no or rarely only minor entanglement of hooks (i.e., 2–3 hooks).

**Table 1:** Gangion treatment descriptions for the experimental longline.

Gangion material	Breaking strength (kg; lbs)	Diameter (mm)	Colour	Average gangion length (cm)	Abbreviation
Monofilament nylon	23; 50	0.70	Clear	42	E-23M
Monofilament nylon	46; 100	0.95	Clear	44	E-46M
Monofilament nylon	91; 200	1.40	Clear	42	E-91M
Multifilament braided nylon	91; 200	2.54	Blue	41	C-91BN

**Figure 1:**
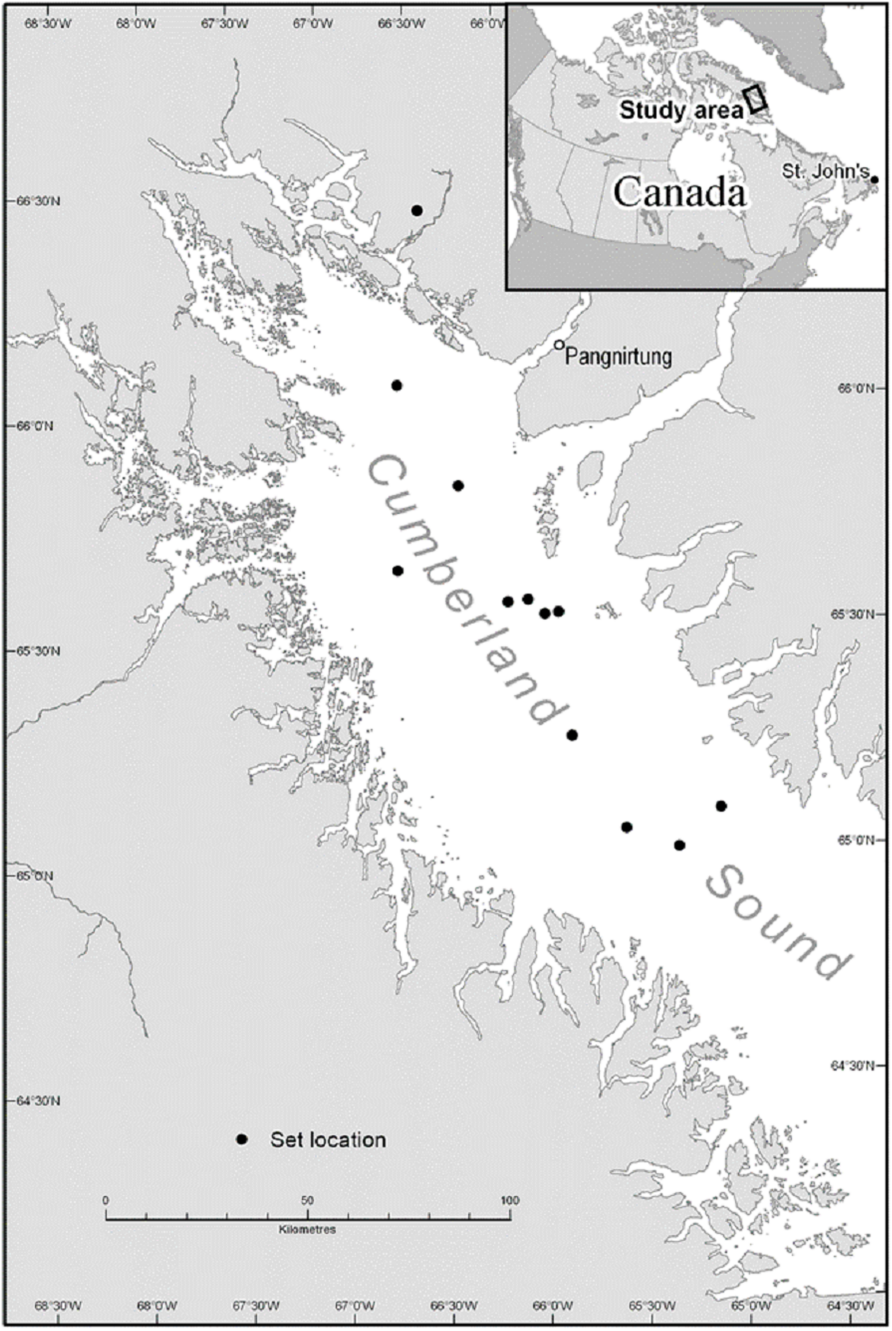
Approximate longline set locations in Cumberland Sound, Nunavut. Note, the accuracy of locations shown is limited by the lack of reliable cartographic data for this region (e.g., one set location is positioned on land despite accurate GPS coordinates indicating a location within the Sound).

A total of 12 experimental longline sets were carried-out in Cumberland Sound between 19 August and 7 September 2012 (Fig. 1). Fishing was conducted from the *M. Nuliajuk*, a 19.5 m Nunavut research vessel that was crewed by experienced Greenland halibut longline fishers. The longline experiments were carried-out together with an annual longline stock assessment survey for Greenland halibut with set locations for both experimental and research survey longlines chosen randomly within defined depth strata (i.e., 600–800, 800–1,000, 1,000–1,400 m). Mean depth at the set locations ranged from 708 to 1,267 m. All longlines were set early in the evening between 18:00 and 21:00 h. Eleven sets were hauled the following morning, with soak time ranging from 12 to 14.25 h (mean, 13.0 h). Due to inclement weather, a single set was not hauled for 36.25 h.

A catch label was assigned to each hook upon haul back of the experimental longlines. Catch labels of interest here were Greenland shark, Greenland halibut, depredated Greenland halibut and hook loss. Greenland halibut were measured for total length (±1 cm) and released. Greenland shark were assigned to one of three total body length size categories (<3 m, 3–4 m and >4 m). Although poorly understood, these size categories approximate the size at maturity in males (3 m) and females (>4 m) (Yano, Stevens & Compagno, 2007; MacNeil et al., 2012). Greenland shark sex, health status (i.e., alive or dead, presence of external gross trauma in the form of lacerations or hemorrhaging), mode of capture (i.e., hooked by the jaw alone, hooked by the jaw and entangled within the mainline, or entangled only). The number of hooks entangled around the body and/or caudal peduncle and caudal fin, time required to disentangle and release a shark, and time required to disentangle the mainline once a shark was released were also recorded. The number of hooks entangled by a shark is a proxy for the length of mainline entangled (i.e., two hooks approximates 3.6 m). Damaged and lost hooks and damaged gangions were replaced with the appropriate fishing line.

The number of hooks used in the experimental longline was approximately 2.5× that used in longline sets in the Cumberland Sound winter fishery for Greenland halibut. However, longline strings with several more hooks (1,000–2,500) are commonly set in open water Greenland halibut fisheries (Young, 2010) and over 5,000 hooks may be fished per day. Gangion length and interval was similar to that used in commercial longline fisheries for Greenland halibut. In offshore open water longline fisheries, circle hook size ranges from 14/0 to 16/0 while 12/0 to 14/0 hooks are typically used in the Cumberland Sound winter fishery. The braided multifilament nylon fishing line is the traditional material used in gangions in Greenland halibut longline fisheries, with breaking strength ranging from 91 to 205 kg (i.e., 200–450 lb). Rotor swivels are not generally used in Greenland halibut bottom longline fisheries. Rather, the gangion is simply tied to the mainline. In the current study, rotor swivels were used to help maintain the 20 gangion replicates of each treatment and to maintain accurate intervals between gangions on the mainline. In open water Greenland halibut longline fisheries the gear is typically left to soak for one night (i.e., 12–20 h).

### Additional data: research survey longlines

The longline experiments were carried out together with an annual Fisheries and Oceans Canada longline stock assessment survey for Greenland halibut that commenced in Cumberland Sound in 2011. Stock assessment survey longlines consisted of 200 circle hooks with the same specifications as those used in the experimental longlines. Further, bait type and size as well as gangion length, interval, and method of attachment to the mainline (i.e., rotor swivels) were the same as the experimental longline. However, gangions in the stock assessment survey longlines were all made from braided multifilament nylon with a 159 kg breaking strength. Hook status categories recorded at haul back were the same as those recorded for the experimental longlines. However, only catch results with regard to Greenland shark, hook loss, and Greenland halibut that were depredated by Greenland sharks are presented here.

Stock assessment survey longlines were attached to and separated from the experimental longline by a 10–15 m length of rope. A single stock assessment survey longline was fished during the first two sets of the experimental longlines (i.e., 600 hooks total; 200 survey longline + 400 experimental longline). Two stock assessment survey longlines were fished thereafter (i.e., 800 hooks total), with a survey longline string attached to each end of the experimental longlines (i.e., the sequence was survey longline, experimental longline, survey longline). In the current study, the total number of hooks in a string was below the number typically used in the open water Greenland halibut fisheries (i.e., 1,000–2,500) and therefore not expected to result in higher catch rates of Greenland shark due to greater dispersion of the chemical bait plume.

### Data analysis

In the experimental longlines, hook loss and catches of Greenland shark and Greenland halibut were combined by longline set for each gangion treatment and expressed as a number per 100 hooks, as a consistent unit of effort. All catch and hook loss data were log (n+1) transformed to obtain homogeneity of variances (i.e., Levene’s test for homogeneity of variances). One-way ANOVAs were used to test for between gangion treatment differences in mean hook loss, mean length of Greenland halibut, and mean CPUE of Greenland halibut and Greenland shark. One-way ANOVAs were also used to test for differences between length categories in mean number of hooks entangled and mean time required to disentangle Greenland sharks. Post-hoc analyses were conducted with Duncan’s new multiple range test. A paired-comparison t-test was used to test the effect of multifilament gangion breaking strength on hook loss in the C-91BN gangion treatment and multifilament gangions used in the stock assessment survey longlines. Least squared regression analysis was performed to test the number of hooks lost-number of sharks captured relationship in the combined experimental and stock assessment survey longlines and the effect of depth of capture on the number of hooks entangled by Greenland shark. The significance level was set at 0.05 for all analyses. Software used to conduct statistical tests was SPSS^®^ 17.0.0 (IBM, 2010).

This study was reviewed and approved by Memorial University’s Institutional Animal Care Committee (Project # 12-01-SG).

## Results

A total of 17 Greenland sharks and 233 Greenland halibut were captured in the 12 experimental longlines (Table 2). There were an additional 17 Greenland sharks captured in the 22 stock assessment survey longlines (Table 2). Greenland sharks were captured in seven of the experimental longline sets while Greenland halibut were captured in all 12 sets. Although Greenland shark were not captured in experimental longline Sets 2, 8, or 10, Greenland halibut that had been depredated by Greenland shark were captured in these sets (Table 2). In addition, Greenland shark depredated Greenland halibut captured in the stock assessment survey longlines during these three sets and four Greenland sharks were captured in the stock assessment survey longlines during Set 8 (Table 2). Greenland halibut that were depredated by Greenland sharks exhibited characteristic circular bite marks. Overall, these results indicate the occurrence of Greenland shark in at least 10 (83%) of the set locations and that these sharks were feeding on the experimental longlines. Greenland shark were not captured in the experimental or stock assessment survey longlines during Sets 1 or 6 nor were there Greenland halibut captured that were depredated in these sets (Table 2).

**Table 2:** Summary of experimental longline catches of Greenland halibut and Greenland shark and number of hooks entangled summed across all gangion treatments.

	Set number												
	1	2	3	4	5	6	7	8	9	10	11	12	Totals
Soak time (hr:min)	12:15	12:30	12:45	13:45	13:00	12:30	14:00	36:15	12:30	13:00	14:15	12:00	
Mean depth (m)	854	1,143	708	863	1,082	720	960	778	891	1,267	833	1,080	
Experimental longline													
Greenland halibut													
Total	23	9	5	23	9	1	69	30	7	42	2	13	233
Depredated	0	1	0	1	2	0	1	1	0	2	0	0	8
Greenland shark													
Total	0	0	2	1	3	0	2	0	1	0	5	3	17
Dead	0	0	0	0	1	0	0	0	1	0	1	0	3
Hooks entangled													
Shark present	0	0	2	0	15	0	30	0	15	0	14	77	153
No shark present	0	68	0	0	0	0	33	0	0	0	0	0	101
Stock assessment longline													
Greenland shark													
Total	0	0	2	0	1	0	0	4	2	1	5	2	17
Dead	0	0	0	0	0	0	0	1	0	0	1	0	2
Hooks entangled													
Shark present	0	0	3	0	23	0	0	9	93	11	33	56	228
No shark present	29	47	14	26	36	25	32	12	10	43	48	67	389
Greenland halibut													
Depredated	0	1	0	0	0	0	0	2	3	2	1	0	9

**Note:**

Greenland shark catches, number of hooks entangled, and number of Greenland halibut depredated are also shown for the stock assessment longline. Soak time and mean depth is shown for each set.

**Figure 2:**
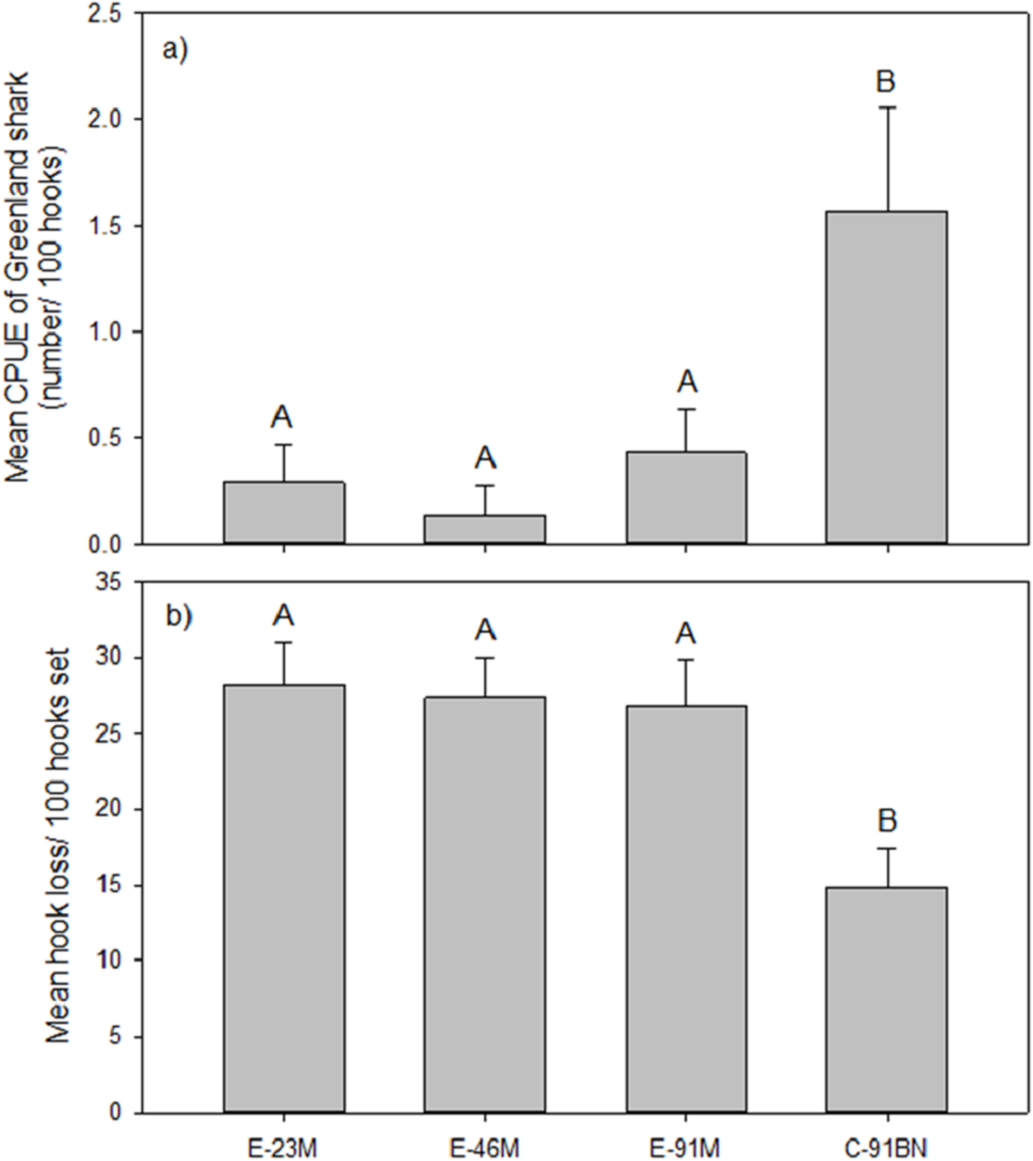
Mean (±1 S.E.) (A) CPUE of Greenland shark and (B) hook loss by gangion treatment in the experimental longline. Homogeneous subsets (A and B) resulting from post-hoc analysis are also shown.

The analysis of gangion treatment effects on mean CPUE of Greenland shark was based on the seven experimental longline sets that captured Greenland shark. Analysis indicated the Greenland shark CPUE differed significantly between gangion treatments in these seven sets (*F*_3, 24_ = 5.399, *p* = 0.006; Fig. 2A). Post hoc analysis indicated there were two homogeneous subsets, with mean Greenland shark CPUE being significantly higher in the braided multifilament gangion treatment than all three monofilament gangion treatments (Fig. 2A). Although the braided multifilament gangion treatment accounted for only 25% of gangions in the experimental longline, it accounted for 65% of Greenland shark captures.

**Figure 3:**
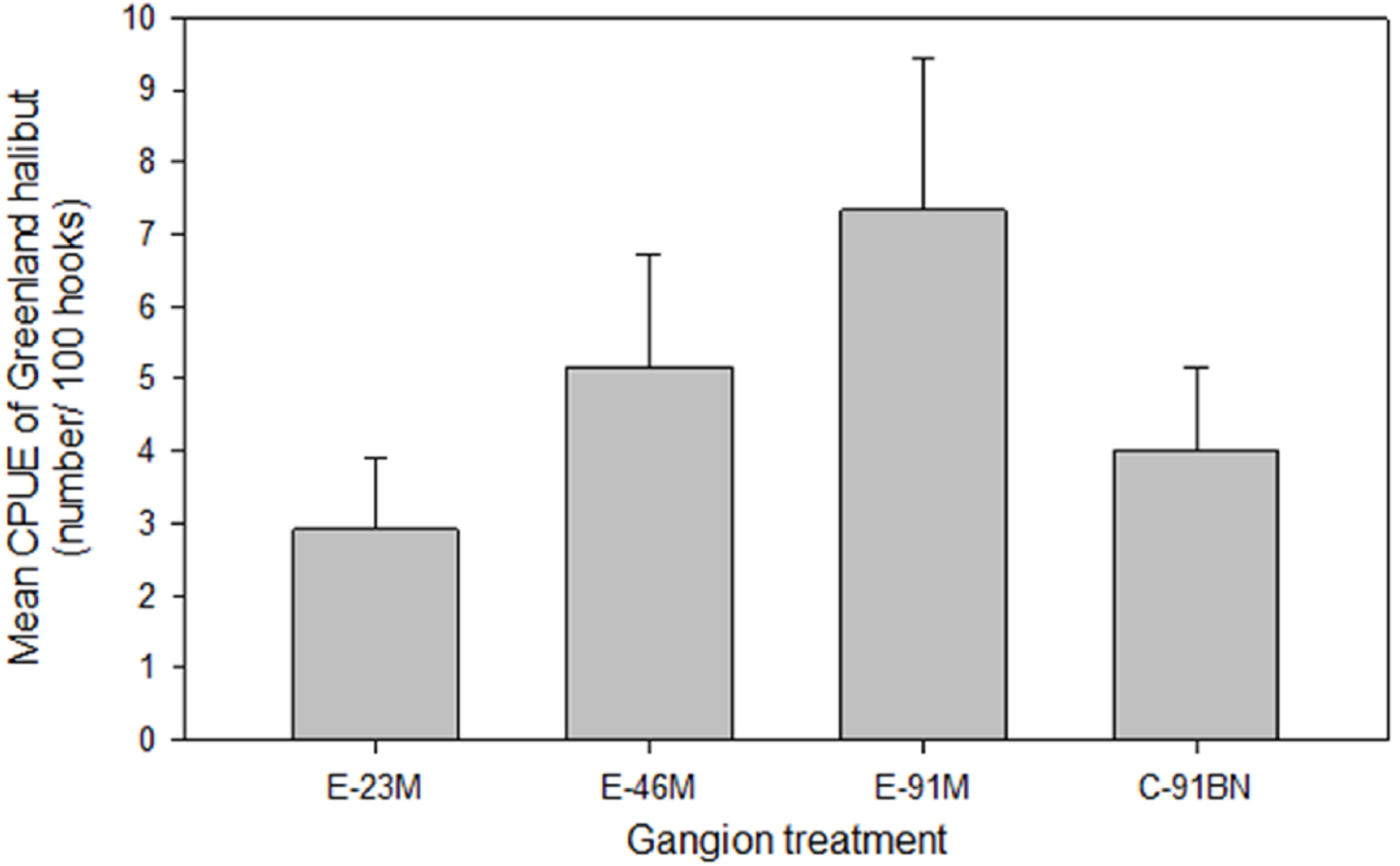
Mean (±1 S.E.) catch per unit effort (CPUE) of Greenland halibut by gangion treatment in the experimental longline.

Analysis indicated there was no significant difference in Greenland halibut mean CPUE (*F*_3,44_ = 1.111, *p* = 0.355; Fig. 3) or mean body length (Table 3) among the gangion treatments.

**Table 3:** Mean (±1 S.E.), minimum, and maximum length (cm) of Greenland halibut by gangion treatment in the experimental longline.

Treatment	Mean length	Min	Max	One-way ANOVA		
				df	*F*-stat	*p*-Value
E-23M	65.2 ± 2.82	50	81	3,229	0.947	0.411
E-46M	67.7 ± 3.17	53	90			
E-91M	66.3 ± 2.92	50	88			
C-91BN	67.0 ± 3.07	47	86			

**Note:**

Results of the one-way ANOVA comparing mean length among gangion treatments is also shown.

**Figure 4:**
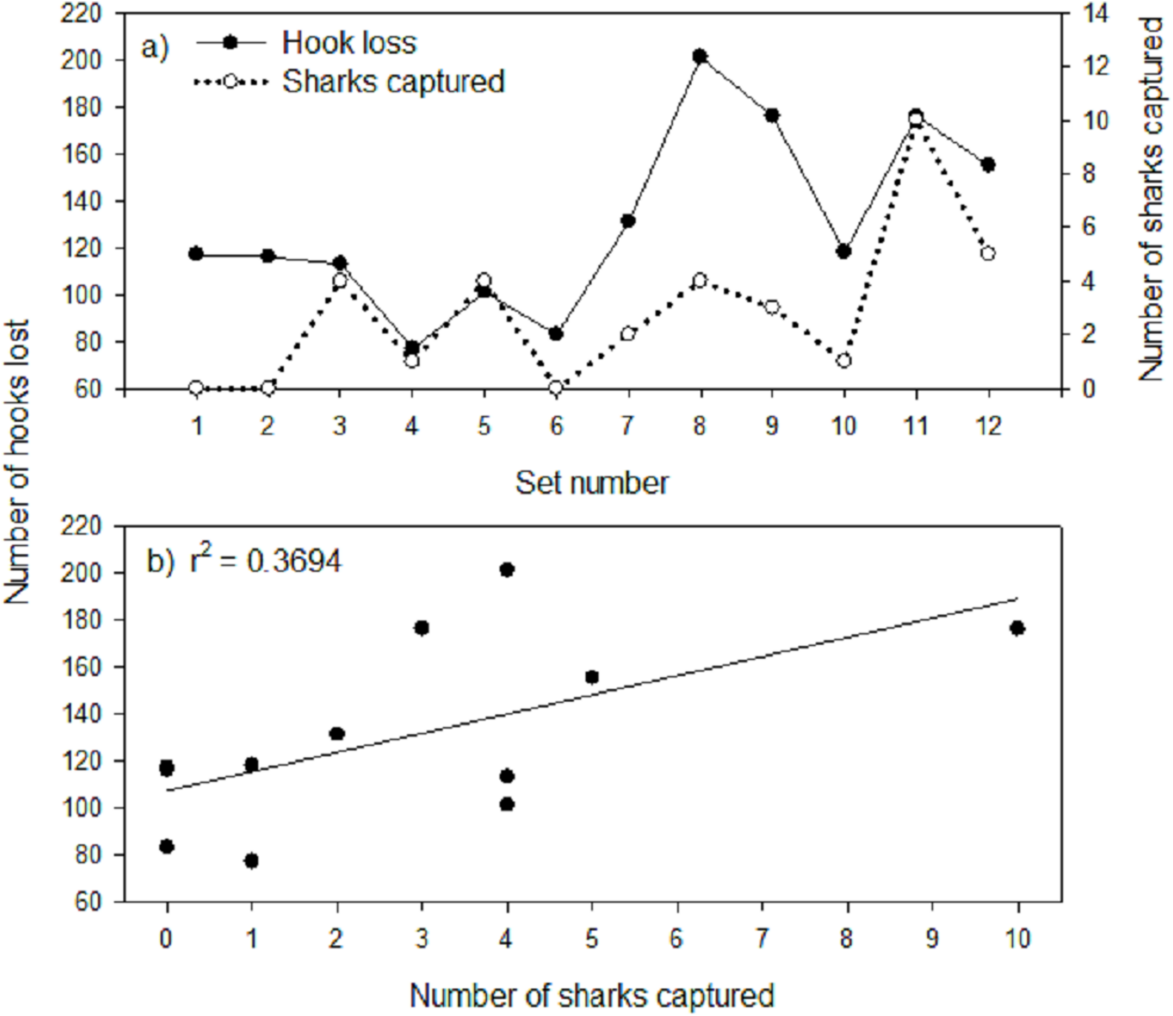
(A) Number of hooks lost and number of sharks captured by set number and (B) number of hooks lost-number of sharks captured relationship for combined experimental and stock assessment survey longline. The line of best fit and coefficient of determination (*r*^2^) are also shown for (B).

Mean hook loss was found to differ significantly among gangion treatments (*F*_3,44_ = 7.180; *p* = 0.001; Fig. 2B). Post-hoc analysis revealed two homogeneous subsets that mirrored the subsets for mean Greenland shark catch rate (Fig. 2B), with 1.8× to 1.9× more hooks lost in the monofilament gangion treatments than in the braided multifilament gangion treatment. A plot of the total number of hooks lost per set in the combined experimental and stock assessment survey longlines demonstrated similar peaks and valleys as the total number of sharks captured (Fig. 4A). Analysis indicated a positive and significant (*p* = 0.036) relationship between the number of hooks lost and number of sharks captured in the combined experimental and stock assessment survey longlines at a set location (Fig. 4B). However, the *r*^2^-value (0.3694) indicates only a weak correlation (Fig. 4B).

The higher breaking strength braided multifilament nylon gangions used in the stock assessment survey longline exhibited a lower mean hook loss (i.e.,9.48 ± 1.22 S.E.) than braided multifilament nylon gangions used in the experimental longline (i.e.,14.83 ± 2.57 S.E.). However, analysis indicated hook loss was independent of breaking strength (*t*_16_ = 1.882, *p* = 0.078).

Three of the Greenland sharks captured in the experimental longlines (Sets 5, 9 and 11) and two of the sharks captured in the stock assessment survey longlines (Sets 8 and 11) were dead when hauled to the surface (Table 2). All of the remaining sharks were released alive and observed to swim away. In addition, there was no evidence of external gross trauma to these sharks in the form of hemorrhaging or lacerations. All of the dead sharks captured in this study had been cannibalized by Greenland sharks as the two potential deep diving predators that occur in Arctic and sub-Arctic waters, the sperm whale (*Physeter macrocephalus*) and northern bottlenose whale (*Hyperoodon ampullatus*), occur only in deep offshore waters of Davis Strait and Baffin Bay where their primary prey is squid (COSEWIC, 2011; Davidson, 2016). Further, we did not observe any large predatory toothed whales in Cumberland Sound during this study. For two of the cannibalized sharks, only the caudal peduncle and caudal fin entangled within the mainline were recovered and for a third shark, only the head was recovered. The two remaining sharks were entangled within the mainline and large circular pieces of flesh had been removed from the body. Overall, 15% of the Greenland sharks captured in the combined experimental and stock assessment survey longlines were cannibalized. Most of these sharks (80%) were cannibalized in overnight sets with an average soak time of 13.2 h (Table 2).

Given the incidence (15%) and extent of Greenland shark cannibalism in the current study it is conceivable that sharks, particularly entangled sharks, could have been cannibalized to the point that their remains were not recovered in the longline gear. During the current study there were many cases where the mainline was entangled, accounting for five to 30 hooks, yet no sharks were present (Table 2). These entanglements were more prevalent in the stock assessment survey longline (i.e., 26 cases) compared to the experimental longline (i.e., eight cases).

The mainline was wrapped around the body and/or caudal peduncle and caudal fin of 76% (25/33) of the Greenland sharks captured that could be assessed in the combined experimental and stock assessment survey longlines (i.e., only the head was recovered for one shark). Entangled sharks accounted for 67% (4/6) of the sharks captured in the three monofilament gangion treatments, 64% (7/11) of sharks captured in the experimental braided multifilament gangion treatment (i.e., C-91BN) and 82% (14/17) of sharks captured in the stock assessment survey longlines. Overall, 84% (26/31) of the sharks that could be assessed were hooked by the jaw. Most (77%, 17/22) of the entangled sharks that could be assessed were found to be hooked by the jaw and two of these sharks had an additional hook with a severed gangion retained in the jaw. It was not possible to open the mouth of entangled sharks that were not jaw hooked or any of the jaw hooked sharks to determine whether a hook was retained in the buccal or pharyngeal cavity. Four of the five entangled sharks that were not hooked by the jaw were captured in the stock assessment survey longline and one was captured in the E-91M gangion treatment.

The behavior of the Greenland sharks captured during this study could be characterized as lethargic and none of the sharks exhibited resistance during haul back or at the surface of the ocean whether they were hooked by jaw alone or when entangled within the mainline. When summed across all monofilament gangion treatments, neither the number of hooks entangled (*t*_10_ = 0.123; *p* = 0.452) nor the time required to disentangle a Greenland shark (*t*_6_ = 0.939; *p* = 0.192) differ significantly among the monofilament and braided nylon gangion treatments in the experimental longline. When combined over both the experimental and stock assessment survey longlines the number of hooks entangled by individual Greenland sharks ranged from 3 to 57 (mean, 16.5 ± 3.57 S.E.) and crew members required 1–79 min (mean, 11.9 ± 4.1 S.E.) to fully disentangle these sharks. It required an additional 1–49 min (mean, 12.3 ± 3.6 S.E.) for 2-3 crew members to disentangle the mainline. An entanglement of 3-57 hooks corresponds to approximately 5.4 m to 102.6 m of mainline (mean, 28.7 ± 5.8 S.E.). A regression analysis that included all entangled Greenland sharks captured during this study (i.e., survey and experimental longlines) revealed the number of hooks entangled by a Greenland shark was independent of depth of capture (*p* = 0.098).

Because of their large body size, none of the Greenland sharks were hauled on board the vessel during disentanglement. Entangled sharks were partially hauled out of water so that a rope could be tied to the mainline below the entanglement and subsequently tied off to remove the strain from the mainline that was wrapped around the shark. At this point the shark was lowered into the water with most of the body submerged. Prior to disentangling, all hooks were removed (i.e., gangions were cut) to avoid injury to the shark and fishers. All sharks were completely disentangled prior to release (i.e., there was no trailing gear embedded in or wrapped around the body, caudal peduncle, or caudal fin). In two cases the mainline required cutting in several locations to facilitate release. It was not possible to remove hooks that were embedded in the jaw and for jaw hooked sharks the gangion was cut at the hook attachment.

Twenty-nine of the Greenland sharks captured in the combined experimental and stock assessment survey longlines were assigned to one of three length categories and sex was recorded for 26 sharks (Table 4). Greenland sharks >4 m in length comprised 21% of the catches, with sharks in the <3 m and 3–4 m length categories nearly equally represented. There was a gradual increase in the percentage of sharks entangled with an increase in body length. One-way ANOVAs indicated the number of hooks entangled by Greenland sharks was independent of body length category (*F*_2,18_ = 0.036, *p* = 0.965) as was the time required to disentangle the sharks (*F*_2,18_ = 0.366, *p* = 0.699). The sex ratio was uneven within the <3 m and >4 m length categories but 1:1 when all length categories were combined (Table 4).

**Table 4:** Catches, across all longline fishing gear, of Greenland shark for three body length categories.

Length category	Number examined	Percent entangled	Sex			Sex ratio (male:female)
			Male	Female	Undetermined	
<3 m	11	64%	3	7	1	0.4:1
3–4 m	12	75%	5	5	2	1:1
>4 m	6	83%	5	1	0	5:1
Total	29		13	13	3	1:1

**Note:**

Percent entangled, sex, and sex ratio are also shown for each length category. Length was estimated for 29 Greenland sharks but sex was only be determined for 26 of these individuals.

Because it is illegal to remove fins from sharks captured in Canadian waters (Fisheries & Oceans Canada, 2007) and it is time consuming to disentangle Greenland sharks captured on longlines, fishers have been observed to release Greenland sharks with trailing fishing gear (S. Grant, 2014, 2015 personal observation). We use the results obtained here to demonstrate the differences between gangion treatments with regard to the length of mainline with gangions and hooks attached that would be discarded when Greenland sharks are release with trailing fishing gear. To simulate commercial conditions the mean CPUE of Greenland sharks was calculated for each gangion treatment using all 12 experimental longline sets (i.e., C-91BN = 0.915 sharks/100 hooks; E-91M = 0.254 sharks/100 hooks; E-46M = 0.083 sharks/100 hooks; E-23M = 0.169 sharks/100 hooks). The percentage of Greenland sharks entangled in the current study (i.e., 76%) was then applied to each gangion treatment to obtain estimates of the mean CPUE of entangled sharks (Table 5). The mean length of mainline that would be discarded per 100 hooks set was then calculated for each gangion treatment based on the mean CPUE of entangled sharks and mean length of mainline entangled by sharks (i.e., 28.7 m). The mean number of hooks entangled by Greenland sharks was used to calculate the number of hooks that would be discarded with the mainline. This value was added to the mean number of hooks lost during fishing to obtain an estimate of total hook loss per 100 hooks set (Table 5). For comparison between years, the mean CPUE of entangled sharks was also calculated for each gangion treatment by applying the percentage of entangled sharks (48%) captured in Cumberland Sound in 2011 (Grant, Sullivan & Hedges, 2018) to our catch rate estimates (Table 5). The mean number of hooks entangled (34.4) and mean length of mainline entangled (61.9 m) by Greenland sharks reported by Grant, Sullivan & Hedges (2018) was used along with the estimated mean CPUE of entangled sharks to calculate the mean number of hooks and mean length of mainline that would be discarded per 100 hooks set when sharks are released with trailing fishing gear (Table 5).

**Table 5:** Summary by gangion treatment of the mean length of mainline discarded and mean number of hooks lost per 100 hooks set when Greenland sharks are released with trailing fishing gear.

Gangion treatment	Current study					Grant, Sullivan & Hedges (2018)				
	CPUE of entangled sharks	Length of mainline discarded (m)	Number of hooks lost			CPUE of entangled sharks	Length of mainline discarded (m)	Number of hooks lost		
			Discarded with mainline	Gangion severed during fishing	Total			Discarded with mainline	Gangion severed during fishing	Total
C-91BN	0.70	20.1	11.2	14.8	26.0	0.44	27.2	15.1	14.8	29.9
E-91M	0.19	5.5	3.1	26.8	29.9	0.12	7.4	4.1	26.8	30.9
E-46M	0.06	1.7	1.0	27.3	28.3	0.04	2.5	1.4	27.3	28.7
E-23M	0.13	3.7	2.1	28.1	30.2	0.08	5.0	2.8	28.1	30.9

At 1.8 m intervals between gangions, a 100 hook section of the mainline would be equivalent to 180 m. In the braided multifilament gangion treatment, the estimated mean length of mainline that would be discarded with entangled Greenland sharks (i.e., 20.1 m; Table 5) would account for 11.2% of the mainline compared to 1.2–3.1% (2.1–5.5 m) in the monofilament treatments. Grant, Sullivan & Hedges (2018) reported a lower percentage of Greenland sharks entangled in longlines. However, because more hooks were entangled, an estimated 15.0% of the mainline would be discarded in the braided multifilament gangion treatment compared to 1.4–4.1% in the monofilament treatments. In addition, because hooks are evenly spaced along the mainline, more hooks will be discarded with the mainline when using braided multifilament gangions. As such, the total number of hooks lost increases and is comparable to the value reported for the monofilament gangion treatments (Table 5).

## Discussion

This study has demonstrated that by changing gangion material in bottom longlines from braided multifilament nylon to monofilament nylon, catch rates of Greenland shark can be significantly reduced without significantly influencing catch rates or mean body length of Greenland halibut. Gangion material also influenced mean hook loss with significantly more hooks lost in the monofilament gangion treatments. Mean hook loss in the monofilament gangion treatments was not only independent of breaking strength but also exhibited very little change with a sequential doubling of the breaking strength. These results suggest a common factor was contributing to hook loss in the monofilament gangion treatments. In pelagic longline fisheries, hook loss on monofilament nylon gangions is attributed to shark bite-offs (Berkeley & Campos, 1988; Branstetter & Musick, 1993; Afonso et al., 2012). However, in bottom longline fisheries, the source of severed gangions and hence hook loss can be caused by several factors. Thus, the higher hook loss in the monofilament gangion treatments cannot be attributed solely to interactions with Greenland sharks.

There is no way to determine how many Greenland sharks were present at each set location or the number of encounters sharks had with hooks during a longline set. However, every hook was in contact with the seabed, increasing the likelihood that most of the hook loss events in both the monofilament and multifilament gangion treatments resulted from gangions severed by hooking on the seabed. For example, the gradual increase and magnitude of hook loss after Set 6 could be caused by the cumulative effects of wear and tear on the gangions over time (Fig. 4) including damage that is not readily visible to the naked eye (e.g., stretching and weakening from hooking on the seabed during haul back). In addition, setting gear on predominantly hard or soft bottom can account for the high variability in hook loss among sets and may account for the high level of hook loss during Sets 1 and 2 when no sharks were captured (Fig. 4). Longline set locations were randomly distributed over a wide range of depths (Table 2) with a minimum 3 km buffer between locations within known depth strata which led to high variability in substrate type among set locations. Capture of soft corals attached to small rocks and cobble as well as presence of straightened hooks were indicative of setting on hard bottom. Ultimately, the method used to select longline set locations likely inflated the overall rate of hook loss relative to what would be expected in commercial longline fisheries for Greenland halibut that tend to target homogenous substrates (i.e., soft mud-sand bottom) and areas of low seabed relief.

In summary, we suspect that the majority of the hook loss events can be attributed to a combination of seabed conditions at the set locations and wear and tear of the gangions with increased susceptibility to severing of the narrower diameter and single strand monofilament fishing line compared to the nine strand braided multifilament fishing line. As such, an increase in severed gangions in the monofilament gangion treatments may be expected. The question is, does the cost of replacing hooks and gangions lost when using monofilament fishing line outweigh the benefits of a reduction in catch rates and entanglement of Greenland sharks? Because a high percentage of Greenland sharks entangle in bottom longline gear, the economic benefits of significantly fewer sharks on monofilament gangions will include: a reduction in the amount of mainline that will be damaged or discarded when releasing entangled sharks, an overall decrease in amount of time associated with releasing sharks, and a reduction in gear loss due to releasing entangled Greenland sharks with trailing fishing gear.

Previous studies have indicated Greenland shark entangle in bottom longlines set for Greenland halibut (Pike, 1994; Young, 2010; Dennard et al., 2010) but only recently has the extent of entanglement been documented (Grant, Sullivan & Hedges, 2018). Compared to the current study, Grant, Sullivan & Hedges (2018) found that a lower percentage of captured Greenland sharks were entangled in bottom longlines (i.e., 48%) and although the mean amount of time required to disentangle sharks was marginally lower (i.e., 9.8 min) the mean number of hooks entangled was substantially higher (i.e., 34.4 hooks vs. 16.5 in the current study). Grant, Sullivan & Hedges (2018) also found that the number of hooks entangled by Greenland sharks and time to disentangle and release sharks increased significantly with body length. No similar relationships were observed in the current study. The absence of similar relationships may be related to the use of broad body length categories and capture of larger or smaller individuals within a length category from year-to-year. In addition, Greenland shark make vertical excursions into the water column (Skomal & Benz, 2004; Stokesbury et al., 2005; Fisk, Lyderson & Kovacs, 2012; Campana, Fisk & Klimley, 2015) and have been observed at the surface of the ocean depredating Greenland halibut captured on longlines (Grant, Sullivan & Hedges, 2018). Thus, annual variability in the vertical capture location within the water column may influence these relationships. Lastly, variability in timing of capture during a set may also be important as sharks captured early in a set may be expected to entangle more hooks than sharks captured a few hours prior to haul back. Hook time recorders have been used to determine when sharks are captured on longlines (Kerstetter & Graves, 2006). However, results for Greenland sharks captured on hook timers will need to be interpreted with caution as Greenland sharks depredate Greenland halibut and hook time recorders that are triggered by Greenland halibut that are consumed by Greenland sharks at the seabed or in the water column during haul back will be misinterpreted as Greenland sharks when the depredating sharks are captured.

The International Union for the Conservation of Nature has listed the Greenland shark as near threatened on the basis of possible population declines and limiting life history characteristics (International Union for the Conservation of Nature (IUCN) (IUCN, 2014)). Bycatch and entanglement of Greenland sharks on bottom longlines is unavoidable and when Greenland sharks are captured efforts should be made to release them in a manner that causes the least harm and increases the likelihood of post-release survival (Food & Agriculture Organization of the United Nations (FAO), 2011). Shark finning, the practice of removing fins, including the caudal fin of entangled sharks while releasing sharks or when disposing of the carcass at sea, has been illegal in Canada since 1994 (Fisheries & Oceans Canada, 2007). This has led to fishers releasing Greenland sharks with trailing fishing gear (Grant, 2014, 2015, personal observations). Negative effects of trailing fishing gear on post-release survival have been documented for the common thresher shark (Sepulveda et al., 2015) when released with considerably less trailing gear than the mean length of mainline entangled by Greenland sharks reported here and by Grant, Sullivan & Hedges (2018). Given the high percentage of entangled Greenland sharks and high mean length of mainline entangled by sharks during the current and past studies (Grant, Sullivan & Hedges, 2018), it is recommended that every effort be made to remove trailing fishing gear from Greenland sharks prior to release.

In the current study, all trailing fishing gear was removed from Greenland sharks. All Greenland sharks that were not cannibalized survived from the time of hooking or entanglement to gear retrieval and disentanglement. This includes sharks that were entangled and required up to 79 min to disentangle owing to the extent of entanglement and inclement weather conditions during haul back. Similarly, Grant, Sullivan & Hedges (2018) found that all entangled sharks captured on longlines in overnight sets of 14–16 h survived the disentanglement process. The Greenland shark’s lack of resistance during haul back and lethargic behaviour observed while at the surface of the ocean in the current and previous studies (Bigelow & Schroeder, 1948; Idrobo & Berkes, 2012; Watanabe et al., 2012; Grant, Sullivan & Hedges, 2018) facilitates disentanglement, prevents injuries to sharks and fishers, and may lead to a high probability of post-release survival when sharks are released with no or minimal trailing fishing gear.

In the current study, we were careful not to harm the sharks and only cut the mainline when it was clear that it could not otherwise be unraveled from the shark’s body or tail region. However, if fishers in high volume-oriented commercial fisheries are expected to completely disentangle Greenland sharks they will undoubtedly cut the mainline as it would be too time consuming to unravel these sharks without doing so. For example, in open water longline fisheries for Greenland halibut 2,500–5,000 hooks are typically tended daily. At a fishing effort of 2,500 hooks/day and estimated mean CPUE of 0.19 entangled sharks/100 hooks in the E-91M gangion treatment this would equate to a mean bycatch of 4.75 sharks/day compared to 17.5 sharks/day at an estimated mean CPUE of 0.70 entangled sharks/100 hooks in the C-91BN gangion treatment. In both of these scenarios 76% of the sharks are assumed to be entangled as was found in the current study. At a mean of 11.9 min to disentangle sharks while avoiding cutting the mainline it would require approximately 57 min to disentangle sharks captured in the E-91M treatment and close to 3.5 h (208 min) for sharks captured in the C-91BN treatment. Even 57 min would be an intolerable period of time to pause the hauler to disentangle sharks in commercial fisheries, especially during inclement weather, or in the presence of whales which are known to depredate Greenland halibut in offshore waters (Dyb, 2006; Mesnick et al., 2006). As such, fishers would make every effort to expedite the process. This would undoubtedly include cutting and discarding sections of the mainline while releasing entangled sharks. Thus, whether entangled sharks are released with or without trailing fishing gear there is a clear economic incentive to mitigate the capture of Greenland sharks by switching from braided multifilament to monofilament gangions.

Rotor swivels were used throughout this study and the use of rotor swivels in bottom set longline fisheries has been found to improve catch rates of targeted species by preventing twisting or tangling of the gangion around the mainline during haul back (Bjordal & Løkkeborg, 1996). In the current study, use of rotor swivels may have increased catch rates of Greenland halibut as they exhibit strong resistance during haul back. Lack of resistance and lethargic behavior exhibited by Greenland sharks during haul back suggests no similar catch rate advantage from using rotor swivels. However, it is conceivable that the use of swivels can reduce entanglement of Greenland sharks at the seabed when jaw hooked sharks roll.

Hooked sharks are often attacked and consumed by other sharks (Motta & Wilga, 2001) and Greenland shark cannibalism has been previously reported on longline gear (Borucinska, Whiteley & Benz, 1998). In the current study, Greenland shark cannibalism was at times severe suggesting more than one shark was involved and the possibility of gregarious foraging behaviour. Entangled sharks may be more vulnerable to cannibalism as most (80%, 4/5) of the Greenland sharks that were cannibalized in the current study were entangled in the mainline. It is unclear whether the fifth cannibalized shark was entangled as only the head was recovered. Given the extent of cannibalism observed when the longlines soaked for as little as 12–14 h it is conceivable that sharks could be fully consumed or consumed to the point that they drop out of the fishing gear during haul back and therefore go undetected. The significant reduction in capture rates of Greenland sharks in monofilament gangions would be expected to lessen the potential for this type of unaccounted fishing mortality.

Capture of significantly fewer Greenland sharks in the monofilament gangion treatments suggests a greater likelihood of escapement by severing of the gangion. Grant, Sullivan & Hedges (2018) reported the capture of eight Greenland sharks with 1–2 additional hooks with severed gangions retained in the jaw (i.e., 30% of sharks captured) and two sharks captured in the current study had an additional hook with a severed gangion retained in the jaw. Feeding behavior of Greenland sharks described by Grant, Sullivan & Hedges (2018) may provide the mechanism that leads to hooking by the jaw rather than the throat or gut and may also facilitate escapement. Grant, Sullivan & Hedges (2018) describe the feeding behavior of a Greenland shark as it preyed on bait suspended in a pot at a depth of 878 m. This shark exhibited suction feeding with five to eight successive suction events during a feeding bout. This feeding behavior would allow a shark to draw a baited hook off the seabed and into its mouth but the pause and minor exhalent current between repeated suction events of a feeding bout is suspected to cause the hook to become embedded in the jaw. Indeed, 85% of the sharks captured by Grant, Sullivan & Hedges (2018) were hooked by the jaw and 84% of the sharks captured in the current study were also hooked by the jaw. Monofilament fishing line is more rigid than multifilament fishing line which may increase the likelihood of the gangion coming in contact with the teeth during repeated suction events of a feeding bout. Moreover, being thinner and single strand the monofilament fishing line would be easier to sever from contact with the teeth then the braided multifilament gangion. Alternatively, it is conceivable that in jaw hooked sharks the monofilament gangion will chafe and sever more readily from contact with placoid scales on the head and body of the shark. The placoid scales of sharks have the same structure as a sharks teeth and the outer enamel layer of placoid scales has been found to have the same hardness as steel on Mohs scale (Moss, 1977).

Given the level of hook loss observed in the current study, hook ‘bite-offs’ by Greenland sharks that result in hooks becoming retained in the throat or gut cannot be ruled out. Borucinska et al. (2002) describe the pathology associated with blue sharks (*Prionace glauca*) that were hooked in the throat and gut. None of the blue sharks examined suffered from debilitating disease and it is unclear whether hooks retained in the throat or gut lead to mortality particularly when gangions are severed by sharks prior to haul back. Borucinska et al. (2002) did not report on the type of hook involved and it is conceivable that when Greenland sharks bite-off and swallow circle hooks with 0° offset that they are less likely to become embedded within the digestive system than J-hooks or offset circle hooks.

Monofilament fishing line is a thin, typically clear material, which is less visible than braided multifilament nylon. It has been suggested that this decrease in visibility explains the increased catch rates of some fish species (Bjordal & Løkkeborg, 1996; Stone & Dixon, 2001; Ward et al., 2008). For example, catch rates of cod and haddock have been shown to be as much as three times higher on monofilament compared to multifilament gangions. It is thought that fish have a harder time identifying monofilament compared to multifilament fishing line and are more apt to prey upon baited hooks on monofilament gangions (Bjordal & Løkkeborg, 1996; Stone & Dixon, 2001). Stone & Dixon (2001) suggested that some species of fish are even able to distinguish thicker and dark coloured multifilament fishing lines easier than monofilament during periods of darkness. The ability of the Greenland shark or Greenland halibut to detect multifilament fishing line easier than monofilament will depend in part on the role that vision plays as a key sensory mechanism during predation. In Arctic waters, the Greenland shark is thought to have severely limited vision (Borucinska, Whiteley & Benz, 1998; Benz et al., 2002; MacNeil et al., 2012) owing to the high prevalence of infestation by the ocular ectoparasitic copepod *Ommatokoita elongata* (Grant, 1827; Berland, 1961). Thus, a change in gangion visibility is not expected to affect Greenland shark depredation rates on baited hooks.

The role of vision in predation by Greenland halibut in Arctic waters at the depths fished in the current study is unclear. If Greenland halibut have the ability to detect multifilament fishing line easier than monofilament then higher catch rates on the latter may be expected. In the current study, the mean catch rate of Greenland halibut on the monofilament gangion treatment with the highest breaking strength (i.e., E-91M) was 1.8× higher than the braided multifilament treatment of equal breaking strength. We speculate that our inability to detect a significant difference in catch rates among gangion treatments was influenced by the low availability of Greenland halibut at the set locations. The set locations for this study were chosen at random and based on depth strata as part of a stock assessment survey, not on known commercial fishing grounds. As such, one set yielded a total catch of 69 Greenland halibut (i.e., 17.3 fish/100 hooks), the maximum recorded, while six sets each yielded less than ten Greenland halibut (Table 2). For comparison, in the Cumberland Sound winter fishery sets as short in duration as 1 h captured a mean of 12 Greenland halibut/100 hooks (Pike & Mathias, 1995). Future studies should seek to determine whether the CPUE of Greenland halibut can be improved significantly by switching gangion material from braided multifilament to monofilament fishing line. The financial incentive of a significantly higher CPUE on monofilament gangions is more likely to facilitate a changeover in gangion material within the fishing industry (Eayers & Pol, 2018) and ultimately reduce the risk of incidental capture of Greenland shark through and overall reduction in annual fishing effort required to capture a given Greenland halibut quota.

## Conclusions

Application of monofilament nylon gangions was beneficial for reducing bycatch impacts on Greenland sharks and were demonstrated to reduce time and gear loss to fishers over that of multifilament gangions without negatively affecting the Greenland halibut catch. Longline fisheries with Greenland shark bycatch are encouraged to replace multifilament gangions with monofilament gangions to reduce ecosystem effects from fishing and reduce the loss of gear and time to fishers. Greenland sharks were found to commonly entangle in bottom set longlines and at times entanglement was substantial. Nevertheless, entangled sharks were found to survive the disentanglement process and observed to swim away. Given the negative effects of trailing fishing gear on post release survival of some species of sharks (Sepulveda et al., 2015) and the high average length of bottom set fishing gear entangled by Greenland sharks in the current and previous studies (Grant, Sullivan & Hedges, 2018), fishers should be encouraged to remove trailing fishing gear from Greenland sharks prior to release. This study shows that the extent of cannibalism upon Greenland sharks captured on longlines can be substantial and raises the possibility of unaccounted mortality which may be mitigated by lower catch rates on monofilament gangions. The capture of substantially more Greenland halibut on monofilament gangions compared to multifilament gangions of equal breaking strength warrants additional studies comparing the capture efficiency of these two gangion materials.

The most controversial issue with regard to switching from multifilament to monofilament gangions is the significant increase in hook loss on the latter and whether gangions severed by Greenland sharks lead to unaccounted fishing mortality. The concern is the potential negative effects when hooks are retained in the jaw, throat, or gut of sharks. In the current study, and a previous Greenland halibut longline study (Grant, Sullivan & Hedges, 2018), most of the Greenland sharks were not only hooked by the jaw but sharks were also found to have additional hooks with severed gangions retained in the jaw. These findings lead us to conclude that Greenland shark depredation on longlines fitted with circle hooks are more likely to lead to hooking in the jaw rather than by the throat or gut and that hooks retained in the jaw do not interfere with feeding.

## Supplemental Information

10.7717/peerj.10407/supp-1 Supplemental Information 1 Greenland shark catch data by gangion treatment. The body size categories, sex, health status, mode of capture, number of hooks entangled, and time required to disentangle sharks is shown.

10.7717/peerj.10407/supp-2 Supplemental Information 2 Greenland halibut catch and length data by gangion treatment.

## Additional Information and Declarations

### Data Availability

The following information was supplied regarding data availability: The raw data are available as Supplemental Files.
